# Validation Study of Kim's Sham Needle by Measuring Facial Temperature: An N-of-1 Randomized Double-Blind Placebo-Controlled Clinical Trial

**DOI:** 10.1155/2012/507937

**Published:** 2012-03-06

**Authors:** Sanghun Lee, Nara Lim, Sun Mi Choi, Sungchul Kim

**Affiliations:** ^1^Division of Standard Research, Acupuncture, Moxibustion and Meridian Research Center, Korea Institute of Oriental Medicine, Daejeon, Republic of Korea; ^2^Department of Acupuncture and Moxibustion, Wonkwang University Oriental Medicine Hospital, Gwangju, Republic of Korea

## Abstract

*Introduction*. In 2008, Kim's sham needle was developed to improve the quality of double-blinded studies. The aim of this study is to validate Kim's sham needle by measuring facial temperature. *Methods*. We designed “N-of-1” trials involving 7 smokers. One session was composed of 2 stimulations separated by a 2 h washout period. Six sessions were applied daily for all subjects. Infrared thermal imaging was used to examine the effects of acupuncture (HT8, KI2) on facial temperature following smoking-induced decrease. *Results*. All subjects demonstrated decreased temperatures after sham needle treatment, but 5 of the 7 subjects showed increased temperatures after real needle treatment. 6 of the 7 subjects showed a significant difference (*P* < 0.05) between treatments with real and sham needles. Thus, the physiological stimulation of Kim's sham needle is different from that of a real needle, suggesting that Kim's sham needle is a potential inactive control intervention.

## 1. Introduction

Experimental studies on acupuncture have been actively conducted to discover new evidence for acupuncture treatment. To facilitate acupuncture research, the Standards for Reporting Interventions in Clinical Trials for Acupuncture (STRICTA) [1] was published in 2002 and updated in 2010. Blind study designs are the most important method for diminishing placebo effect and for reducing bias. Accordingly, Streitberger and Kleinhenz [2] and Park et al. [3] developed sham acupuncture devices. Miyazaki et al. [4] conducted a double-blind clinical trial to compare a press needle to a placebo lacking the needle element. Because of the characteristics of these devices, however, the ability to blind subjects is limited and double-blind studies are difficult to administer. Therefore, few studies have been conducted in compliance with the standard guidelines for placebo-controlled clinical trials in 2008 to address this problem. Fregni et al. [5] claimed that an important challenge in using a placebo is the development of a sham device that has similar appearance and induces a feeling similar to that induced by the real device. A previous study of Kim's sham needle [6] showed that neither subjects nor acupuncturists could visually distinguish the sham needle from the real needle; there was also no significant difference in the judgment of needle type by skin sensation between the groups. We conducted N-of-1 trials involving smokers to validate Kim's sham needle by observing facial temperature changes before and after administration of acupuncture. A smoking-induced drop in the body surface temperature is caused by the contraction of blood vessels. Sarin et al. [7] reported that blood flow to the digital microvasculature was reduced by 42% following smoking. Nadler et al. [8] reported that smoking suppressed the formation of prostacyclin, causing vasoconstriction, and after examining the acute effect of smoking on the microcirculation, Reus et al. [9] found that the contraction of arterioles causes decrease in blood flow. The decreased skin temperature noted 5 min after smoking a cigarette may be associated with this effect. These results were also in agreement with those of West and Russell [10].

In this study, we tested the feasibility of using Kim's sham needle in a double-blind clinical trial of the efficacy of acupuncture to reverse smoking-induced decreases in body temperature.

## 2. Subjects and Methods

### 2.1. Study Design and Subjects

We conducted a randomized controlled trial with a double-blind evaluation process and evaluated the results on the basis of statistical analysis. The study was conducted at the Wonkwang Oriental Medicine Hospital in Gwangju, Republic of Korea, over a period of 2 months. All subjects gave their informed consent before participating in the study. Both the acupuncturists and subjects in the study area received information about the study and inclusion criteria. We also informed them about the procedure for randomizing the acupuncture treatments. All interventions were performed by an acupuncture specialist.

 Seven healthy male smokers who met the inclusion criteria were enrolled in this clinical trial (Table 1). The inclusion criteria were as follows: (1) submission of the written informed consent; (2) lack of exercise within 24 h of stimulation; (3) lack of intake of tobacco, alcohol, green tea, and coffee within 8 h of stimulation; (4) duration of more than 1 h between eating and stimulation.

The exclusion criteria were as follows: (1) subjects with cardiovascular diseases, including hypertension, arrhythmia, and ischemic heart disease; (2) those with endocrine diseases, including diabetes mellitus and thyroid diseases; (3) those with kidney diseases, including chronic renal failure; (4) those with a past history of surgery for diseases, such as gastric cancer; (5) those with febrile diseases, such as epileptic seizures; (6) other cases (e.g., dysautonomia, cancer, or alcoholism).

### 2.2. Randomization and Blinding

The subjects were randomized using a random number table, generated by a randomizer form at http://www.randomizer.org. One set of needles was randomly chosen for use in 1 session of the trial. The set was composed of 2 types of needles, namely, a real needle and a sham needle. The experimental sequence of the needle types was randomly determined by a third party and opened after the all session completely; therefore, both the physicians and subjects were blinded. One session was composed of 2 stimulations separated by a 2 h washout period (Table 2).

### 2.3. Procedure Details

**Figure 1:**
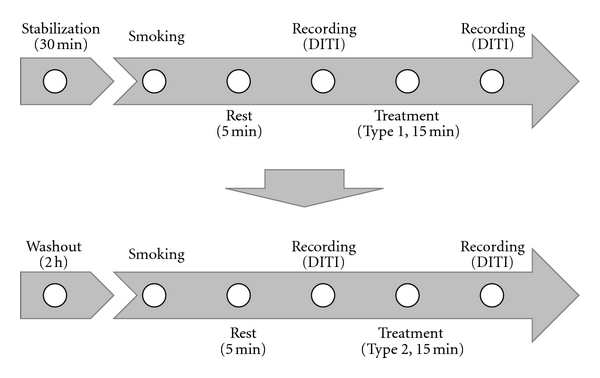
Flow of participants through the trial. The top panel represents the first treatment (either sham or real) that the subject will undergo. This is followed by a 2 h washout followed by the alternate treatment (either real or sham), as shown in the bottom panel.

The experimental procedures were as follows: (1) following a 30 min stabilization period, the subjects were encouraged to smoke tobacco (Dunhill Lights, Republic of Korea). (2) After 5 min (once smoking was finished), the facial temperature was photographed using digital infrared thermal imaging (DITI, Dorex, DTI-16UTI, USA). (3) Either an invasive needle or a noninvasive needle was randomly applied to the subjects. (4) After retaining the needle for 15 min, DITI photography was taken again. (5) Following a 2 h rest, the subjects were encouraged to smoke tobacco. (6) After 5 min (once smoking was finished), DITI photography was taken again. (7) The remaining needles were applied to the subjects. (8) After retaining the needle for 15 min, DITI photography was taken again. All the process was conducted in the sitting position in the DITI studio with constant temperature and humidity. The above procedure was considered 1 pair. 1 pair of procedure was carried out six days with each subject (Figure 1).

#### 2.3.1. Real and Sham Acupuncture

**Figure 2:**
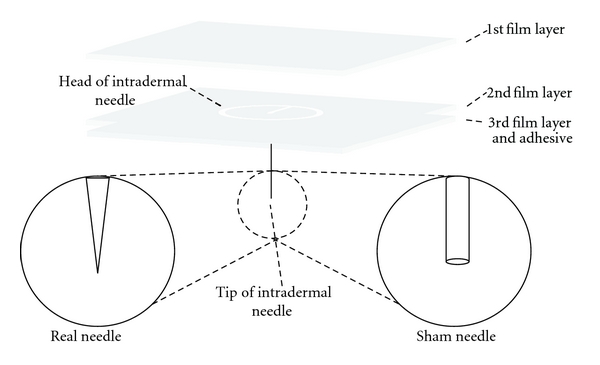
Differences between real needle and sham needle shape. The structure, color, and shape of the sham needle were the same as that of the real needle with the exception of the blunt tip.

**Figure 3:**
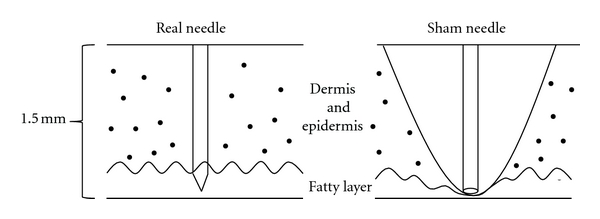
Difference between real needle and sham needle stimulation. While real needle tip is inserted into the epidermis invasively, sham needle tip simply presses the skin surface noninvasively.

The acupuncture intervention entailed the placement of an intradermal T*-*shaped needle (thickness, 0.2 mm; length, 1.5 mm; diameter, 2 mm: model HL-607; Haenglim Seowon Medical, Republic of Korea) for 15 min. The structure, color, and shape of the sham needle (preproduced by Haenglim Seowon Medical) were the same as that of the real needle with the exception of the blunt tip [6]. This blunt tip made it impossible to insert the needle into the skin; however, the tactile sensation was similar to that of the real T-shaped intradermal needle (Figures 2 and 3).

#### 2.3.2. Selection of Acupuncture Points

**Figure 4:**
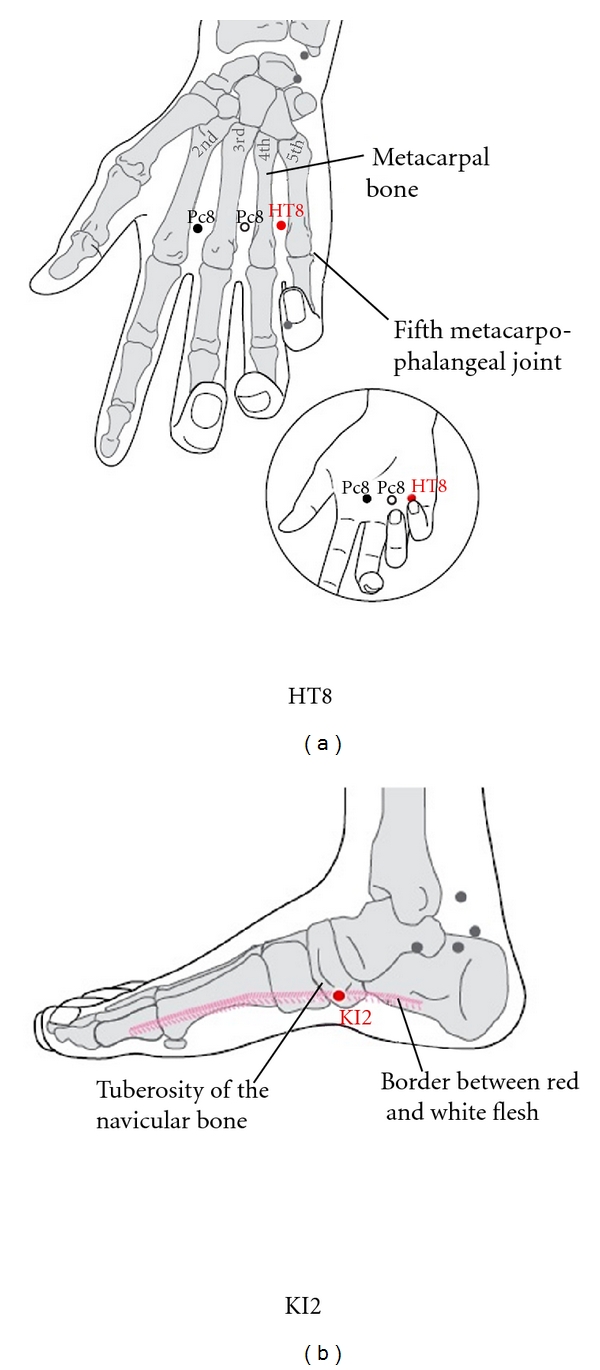
Location of selected acupuncture points for tonification of heart fire. HT8 is used to generate warmth by tonification of the heart fire and KI2 is used to generate warmth by tonification of the kidney fire. The selection of acupuncture points located were based on publication of WHO standard acupuncture point locations [11].

Following the literature of traditional Korean medicine, “Sa-am Five-Element Acupuncture,” the acupuncture points HT8 and KI2 on both the left and right sides of the body were selected to stimulate the blood circulation (Figure 4). These sets of acupuncture points are used to generate warmth by tonification of the heart fire. All operations were performed by the acupuncture specialist according to the WHO criterion for standard acupuncture point locations.

#### 2.3.3. Facial Temperature Measured by DITI

DITI, installed in a photography laboratory for body temperature (DITI studio), was used to measure facial temperature. An infrared photograph of facial temperature was captured such that extrinsic light and heat were blocked and the temperature and indoor atmosphere were homogeneously maintained (24°C ~ 26°C). Beginning 24 h before the experimental procedure, the subjects were instructed to follow these guidelines [12].

(1) No stimulations that provoke changes in the body surface temperature, including physical therapy, drinking, and drug use, were allowed. (2) Two hours prior to photography, the subjects were not allowed to smoke cigarettes. (3) The subjects were encouraged to remain psychologically stable prior to photography. (4) For the adaptation of body surface temperature, the subjects rested in a laboratory room for 20 min. (5) Any activities that affect body surface temperature, such as sunbathing were strictly prohibited.

 A photograph of the body surface temperature of all subjects was captured, with the subjects in a seated position at the same location. The mean temperature was measured by drawing a circle with a diameter that extended from 1 cm above the median point between the eyebrows to 1 cm below the midpoint of the philtrum midline.

#### 2.3.4. Statistics

All statistical analysis was performed by a statistician. A paired sample *t-*test was used to analyze each subject using Microsoft Excel 2010 (Microsoft, USA). A *P* value of <0.05 (two-tailed) was considered statistically significant.

## 3. Results

**Figure 5:**
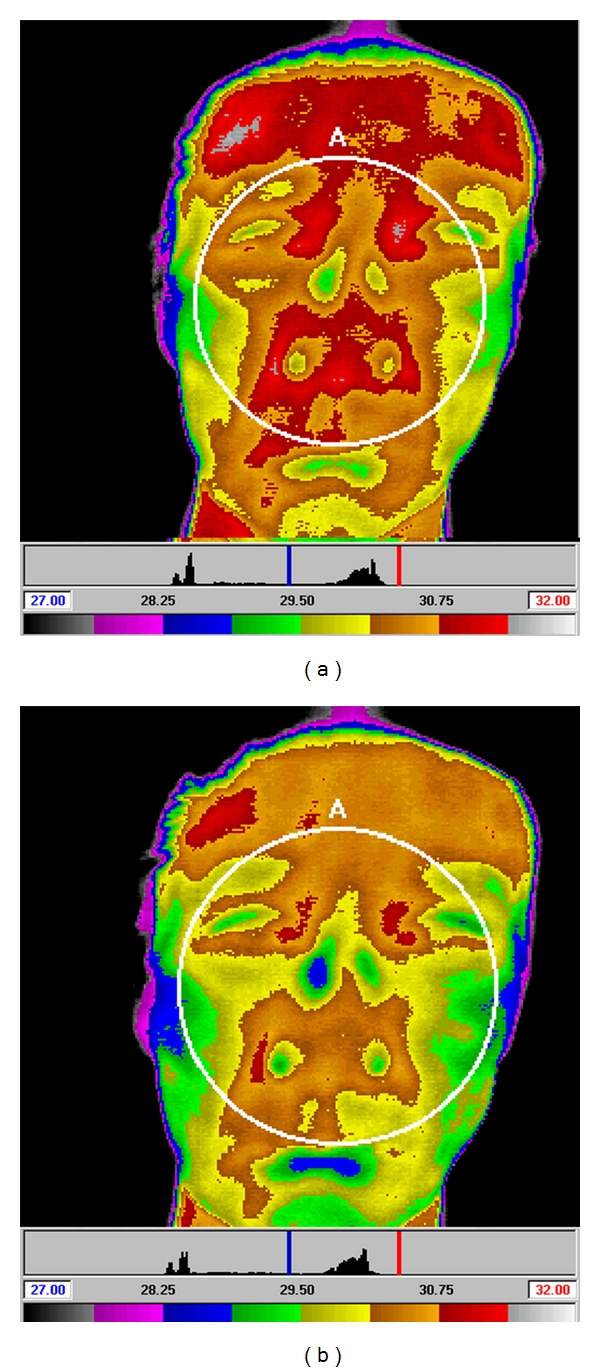
Facial temperature before and after treatment with a sham needle. The changes in facial temperature after smoking (a) and after being treated by sham needle (b). After treatment with sham needle, the average facial temperature shows an overall decrease. On the color bar below the photos, lower temperatures are located on the left.

**Figure 6:**
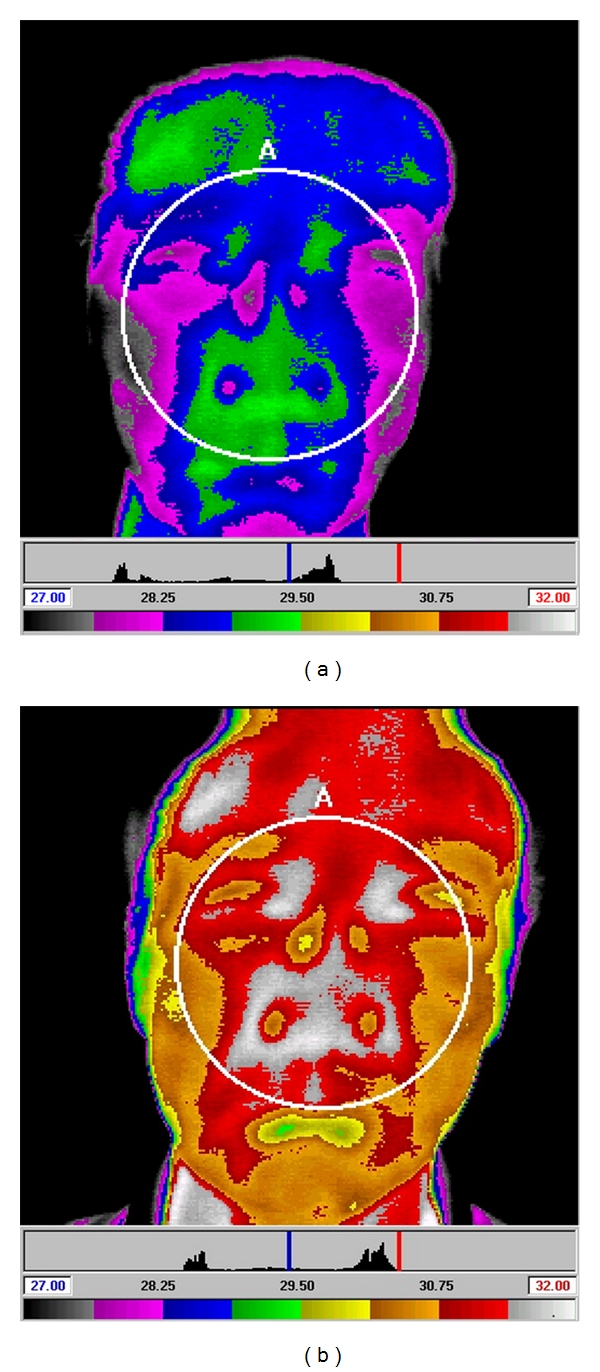
Facial temperature before and after treatment with a real needle. The changes in facial temperature after smoking (a) and after being treated by real needle (b). After treatment with real needle, the average facial temperature shows an overall increase. On the color bar below the photos, higher temperatures are located on the right.

In subjects who were treated with a sham needle following smoking, DITI showed a marked decrease in the facial temperature (Figure 5). In subjects who were treated with a real needle following smoking, however, DITI showed a marked increase in the facial temperature (Figure 6).

**Figure 7:**
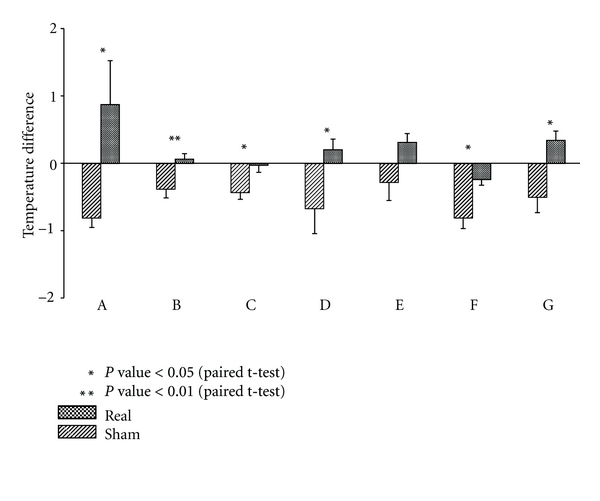
Temperature change before and after treatment with real or sham needle: seven male smokers (A–G) were treated with either a sham needle or a real needle 5 min after smoking and their facial temperature was monitored by DITI. The results show that treatment with a sham needle resulted in an overall decrease in facial temperature, while treatment with a real needle resulted in an overall significant increase in facial temperature in 6 subjects. Values on the graph represent the mean (SD) of 6 independent treatments with 2 h intervals.

Considering the mean values of the differences in facial temperature, all subjects demonstrated decreased facial temperatures after treatment with sham needles, while 5 of the 7 subjects showed increased facial temperatures after treatment with real needles. For 6 of the 7 subjects, a paired sample *t*-test showed a significant difference (*P* < 0.05) in the facial temperature between the treatments with real and sham needles (Table 3 and Figure 7).

At the end of the study, all subjects were asked whether the specific round of acupuncture was invasive or noninvasive. Three of the 7 subjects responded correctly. Physicians could not also differentiate between the 2 acupuncture methods during the acupuncture process.

## 4. Discussion

Although Streitberger and Kleinhenz [2] and Park et al. [3] developed sham acupuncture devices, the ability to blind subjects from experimental manipulations is limited. To reduce the placebo effect and investigate the effect of the treatment, double-blinding is regarded as the gold standard [13]. Kim [6] reported that both lay people and acupuncturists could not discriminate between real acupuncture and sham acupuncture on the basis of the sensation when Kim's sham needle was applied to the acupuncture point LI4. Moreover, in a masking test in the present study, the rate of correct responses did not exceed 50%. This result showed that blinding was successfully conducted in subjects. Unlike a randomized controlled trial, an N-of-1 trial investigates the best treatment for individual patients rather than the best treatment for a group as a whole. Therefore, N-of-1 trials can provide clinicians with data on the best option of treatment for individual patients [14]. On the basis of a theory in traditional Korean medicine called the “Sa-am Five-Element Acupuncture” for increasing skin temperature, we choose HT8 (the fire-acupoint of the fire meridian) and KI2 (the fire-acupoint of the water meridian) bilaterally to stimulate blood circulation. “Sa-am Five-Element Acupuncture” was written by the legendary monk Sa-am Taoyin in 1644; it uses the “Five-Phase” theory as a treat principle [15].

Smoking-induced changes in the peripheral blood flow are primarily mediated by nicotine. This phenomenon is seen in smokers at a lower dose of nicotine through a direct effect on the brain stem or through the activation of the afferent pathways of the chemoreceptors in the central nervous system. The cardiovascular system is also affected, as changes in the peripheral blood vessels result in the constriction of the blood vessels in the skin. On the basis of the findings that *α*/*β*-adrenergic blockages prevented these effects on the cardiovascular system and metabolism, it was suggested that the smoking-induced cardiovascular effects are mediated by the activation of the sympathetic nervous system [16, 17]. After smoking a cigarette, blood flow in digits has been reported to decrease by 42% ± 6% [7]. As shown in the present study, the decrease in the skin temperature noted 5 min after smoking a cigarette may be associated with this effect. These results were also consistent with the results of West and Russell [10], who noted a decrease in the body temperature after smoking. Jensen et al. [18] reported that subcutaneous blood flow decreases by approximately 50% after smoking a cigarette, and that the oxygen partial pressure is lowered in the tissue for approximately 1 h.

After acupuncture treatment with sham needle, facial temperature decreased in all subjects. However, in the group treated with real needle, facial temperature increased in 5 subjects and decreased in 2 subjects. The data from the 7 subjects were analyzed using a paired sample *t*-test, and the results showed that all subjects but 1 had a significant difference in facial temperature. These results indicate that the real needle was more effective than the sham needle in raising facial temperature, which was initially lowered because of smoking. The results showed that the effects of real and sham acupuncture were significantly different in 6 of the 7 subjects. Future research regarding acupuncture and nonacupuncture point treatments with real and sham needles may help garner sufficient scientific evidence to validate Kim's sham needle and increase scientific precision and accuracy in clinical trials on acupuncture.

In conclusion, an N-of-1 trial was performed to validate Kim's sham by measuring facial temperature. Real needle treatment after smoking caused a significant increase in the facial temperature in 6 of 7 subjects compared to Kim's sham needle. This result indicates that Kim's sham needle has a different physical effect on skin temperature. Using Kim's sham needle may facilitate the use of double-blinded acupuncture trials. Further studies using this sham needle might provide more evidence and improve the scientific quality of clinical trials on acupuncture.

## Figures and Tables

**Table 1:** Characteristics of the subjects.

Subject	Sex/age (yrs)	Height (cm)/weight (kg)	Smoking amount (pack)	Smoking duration (yrs)
A	M/56	172/63	1.5	30
B	M/25	168/60	1	2
C	M/26	167/65	0.5	1
D	M/25	173/65	1	3
E	M/31	175/68	0.5	1
F	M/26	183/80	0.5	1
G	M/25	175/70	0.5	1

**Table 2:** Allocation of stimulation.

	A	B	C	D	E	F	G
1	Sham	Real	Real	Sham	Sham	Sham	Sham
	Real	Sham	Sham	Real	Real	Real	Real
2	Real	Sham	Real	Real	Real	Sham	Real
	Sham	Real	Sham	Sham	Sham	Real	Sham
3	Real	Real	Sham	Sham	Sham	Real	Sham
	Sham	Sham	Real	Real	Real	Sham	Real
4	Sham	Sham	Real	Sham	Real	Sham	Sham
	Real	Real	Sham	Real	Sham	Real	Real
5	Sham	Real	Sham	Real	Sham	Real	Real
	Real	Sham	Real	Sham	Real	Sham	Sham
6	Real	Sham	Real	Sham	Real	Sham	Sham
	Sham	Real	Sham	Real	Sham	Real	Real

**Table 3:** Temperature changes after smoking and treatment with real or sham needle.

Subject	Needle	After smoking (°C)	After needling (°C)	Temperature difference (°C)	*P value <0.05
					**P value <0.01
					(paired *t*-test)
A	Real	30.01 ± 0.99	30.89 ± 1.03	0.87 ± 1.60	0.0270*
	Sham	30.71 ± 0.96	29.89 ± 1.20	−0.81 ± 0.35	
B	Real	31.72 ± 0.62	31.78 ± 0.53	0.06 ± 0.20	0.0072**
	Sham	32.04 ± 1.32	31.65 ± 1.41	−0.38 ± 0.33	
C	Real	32.05 ± 0.94	32.01 ± 0.72	−0.03 ± 0.26	0.0138*
	Sham	31.66 ± 0.42	31.23 ± 0.33	−0.43 ± 0.26	
D	Real	31.16 ± 0.92	31.50 ± 0.73	0.20 ± 0.39	0.0164*
	Sham	31.57 ± 0.71	30.90 ± 1.01	−0.67 ± 0.92	
E	Real	31.86 ± 1.10	32.17 ± 1.01	0.31 ± 0.32	0.0688
	Sham	32.49 ± 0.65	32.20 ± 0.37	−0.28 ± 0.67	
F	Real	32.60 ± 0.76	32.36 ± 0.68	−0.24 ± 0.21	0.0450*
	Sham	33.24 ± 0.79	32.42 ± 0.74	−0.81 ± 0.39	
G	Real	32.32 ± 1.03	32.67 ± 0.78	0.34 ± 0.34	0.0431*
	Sham	32.97 ± 0.51	32.47 ± 0.45	−0.50 ± 0.57	
